# Developmental constraints enforce altruism and avert the tragedy of the commons in a social microbe

**DOI:** 10.1073/pnas.2111233119

**Published:** 2022-07-15

**Authors:** Laurence J. Belcher, Philip G. Madgwick, Satoshi Kuwana, Balint Stewart, Christopher R. L. Thompson, Jason B. Wolf

**Affiliations:** ^a^Milner Centre for Evolution, University of Bath, Bath BA2 7AY, United Kingdom;; ^b^Department of Biology and Biochemistry, University of Bath, Bath BA2 7AY, United Kingdom;; ^c^Centre for Life’s Origins and Evolution, Department of Genetics, Evolution and Environment, University College London, London WC1E 6BT, United Kingdom

**Keywords:** cooperation, altruism, tragedy of the commons, enforcement, developmental constraints

## Abstract

Organisms often cooperate through the production of freely available public goods. This can greatly benefit the group but is vulnerable to the “tragedy of the commons” if individuals lack the motivation to make the necessary investment into public goods production. Relatedness to groupmates can motivate individual investment because group success ultimately benefits their genes’ own self-interests. However, systems often lack mechanisms that can reliably ensure that relatedness is high enough to promote cooperation. Consequently, groups face a persistent threat from the tragedy unless they have a mechanism to enforce investment when relatedness fails to provide adequate motivation. To understand the real threat posed by the tragedy and whether groups can avert its impact, we determine how the social amoeba *Dictyostelium discoideum* responds as relatedness decreases to levels that should induce the tragedy. We find that, while investment in public goods declines as overall within-group relatedness declines, groups avert the expected catastrophic collapse of the commons by continuing to invest, even when relatedness should be too low to incentivize any contribution. We show that this is due to a developmental buffering system that generates enforcement because insufficient cooperation perturbs the balance of a negative feedback system controlling multicellular development. This developmental constraint enforces investment under the conditions expected to be most tragic, allowing groups to avert a collapse in cooperation. These results help explain how mechanisms that suppress selfishness and enforce cooperation can arise inadvertently as a by-product of constraints imposed by selection on different traits.

## Significance

Organisms often generate benefits shared among their whole group, but such cooperation is vulnerable to collapse if individuals can instead benefit by exploiting the cooperation of others. While relatedness can promote cooperation, many species lack reliable mechanisms to ensure high relatedness. They are therefore vulnerable to a breakdown of cooperation unless they are able to enforce cooperation. We test this idea through experimental manipulation of group composition in a social microbe. We find that groups avert the expected collapse in cooperation at low relatedness due to inadvertent enforcement of cooperation by a mechanism that prevents errors in multicellular development. Our findings explain how mechanisms that promote cooperation can arise as by-products of natural selection acting on traits in other contexts.

Individuals often perform cooperative acts that are costly to the actor but benefit all members of their group, regardless of individual contributions (1, 2). While this type of cooperation through public goods can be hugely beneficial for the group, the self-sacrifice it requires may outweigh the incremental benefits that an individual’s own contribution adds to group success (3, 4). As a result, individuals will often lack motivation to contribute to public goods, which can lead to the tragedy of the commons (5), where selfish behaviors that maximize personal interests reduce the success of the whole group (4). The potential risk that the tragedy represents to societies has long been recognized in economics (6, 7), and its role in shaping the evolution of cooperation and group organization in nature has become increasingly recognized in biology (3, 8, 9). Despite this, we still have a limited understanding of the conditions under which groups actually suffer from the tragedy, the factors that determine how bad the outcome is, and the mechanisms that mitigate its impact and allow groups to avert the most catastrophic consequences where public goods are not produced at all.

**Fig. 1.**
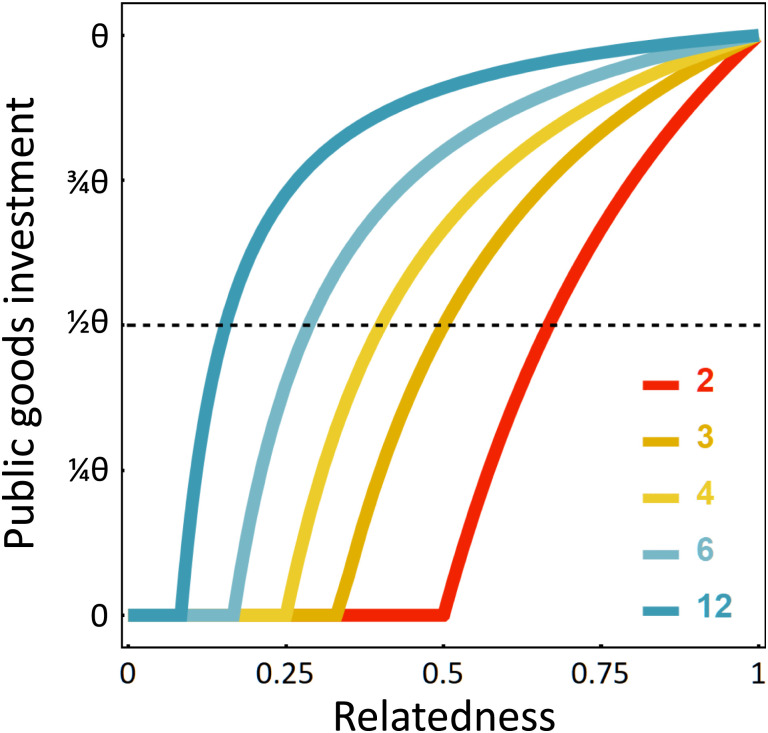
A player’s investment in public goods as a function of their relatedness to the group. The different lines correspond to different benefits relative to costs (*b*/*c*) (see inset legend). Each line corresponds to a value of $\frac{1}{2}\left(\frac{1}{c}-\frac{1}{br_{i}}\right)$ whenever *r_i_* > *c*/*b*, and a value of 0 otherwise. The *y* axis scales investment relative to that which optimizes group fitness (*θ*).

In biological systems, groups can decrease the threat of the tragedy by ensuring that relatedness is high enough to motivate public goods investment (3). Relatedness is a powerful motivator of group-beneficial behaviors at a genetic level because the benefits accrued by copies of the actor’s genes present in groupmates can more than offset the cost of the self-sacrifice by the actor (10). However, many systems lack mechanisms that can always reliably ensure high relatedness (11–13). Reduced relatedness can be problematic for groups because it can shift the balance of selection away from favoring individuals acting for the good of the group toward maximizing their selfish interests. However, under all relatedness conditions, selection should favor individuals who are able to contribute to public goods at the level that maximizes their inclusive fitness (given their relatedness), which can be accomplished by strategically adjusting contributions depending on their relatedness to the group (2, 14–18). The logic of such strategic cooperation is captured in the “Collective Investment” game (17, 18) in which the “players” are different genotypes interacting in groups and each player decides what fraction of their resources to invest into production of public goods. Group members benefit (at a rate given by *b*) from the total collective amount of public goods produced, while individuals pay a personal cost (at a rate of *c* per unit contributed) in terms of reduced direct reproduction or some other component of direct fitness (see *Materials and Methods* for a brief presentation of the model). Given some benefits and costs of public goods production, the optimal level of investment for each player will depend on their relatedness to the group (*r_i_*) (Fig. 1). When a player has a relatedness of 1 to the group (i.e., only one genotype is present in the group), they should invest at a level that maximizes the total fitness of the group (denoted by *θ*, which equals [b−c]/2bc). However, as relatedness declines from 1, motivation to invest in public goods declines, eventually reaching 0 when relatedness drops below the level where the costs outweigh the benefits (which occurs once *r_i_* < *c*/*b*, following Hamilton’s rule) (10). The degree to which this will occur will depend on the level of relatedness within a group and the magnitude of benefits from public goods relative to their costs, which shapes motivation to invest across the range of relatedness (Fig. 1). Furthermore, the catastrophic collapsing tragedy is guaranteed to arise under conditions where no group member is motivated to invest, unless there is some form of enforcement. Indeed forms of enforcement have been reported in nature (3, 14, 19–23), but we still have a very limited mechanistic understanding of their evolution, their mode of action, the role they play in the face of strategic cooperation, and the extent to which they provide a solution to the tragedy of the commons.

To understand the threat posed by the tragedy of the commons in a natural system, and the potential for enforcement to avert its impact, we measured the pattern of relatedness-dependent public goods investment in groups containing multiple strains of *Dictyostelium discoideum* (replicated across several different sets of strains) and used these to estimate the parameters of the Collective Investment game. In this system, single-celled individuals aggregate to collectively form a fruiting body constructed of a stalk (the public good) that facilitates dispersal of spores (the benefit from public goods) (24–26). Data on the frequency of clonality/chimerism in nature indicate that both clonality and chimerism are relatively common (27). Clonality should provide strong selection for a system of canalization (28) that ensures adaptive fruiting body proportioning to optimize clonal group success. Similarly, the high frequency of chimerism in nature [with about a quarter of aggregations containing multiple genotypes (27)] provides ample opportunities for selection to shape adaptive responses to chimerism. Indeed, *D. discoideum* has been shown to exhibit strategic adjustment of contributions to the stalk in response to relatedness (17) and an allorecognition mechanism that can increase relatedness by causing partial segregation in chimeric aggregations (29–31). However, while segregation can potentially limit opportunities for selfishness to undermine group success, it does not allow strains to completely avoid the threat of the tragedy because the extent of segregation varies depending on the genotypes of the interacting strains and, even when high, it does not result in clonality (31). Instead, it simply increases the variance in relatedness across fruiting bodies, which allows some groups to escape the worst of the tragedy by ending up with one strain with high relatedness (which can ensure adequate stalk investment), although many other groups remain highly heterogeneous and potentially suffer the tragedy (31). Moreover, strains with low relatedness to their group show lower levels of segregation (17), which means that groups containing a large number of strains, each with low relatedness, are expected to show relatively low levels of segregation. These patterns of imperfect segregation mean that high relatedness cannot be ensured and, critically, strains can be trapped in the most tragic conditions, where no strain has high enough relatedness to be motivated to invest while strains are able to segregate away from the group. Under these conditions, natural selection could favor processes that enforce cooperation and limit selfish behavior to avert the catastrophic collapse of stalk production.

## Results and Discussion

**Fig. 2.**
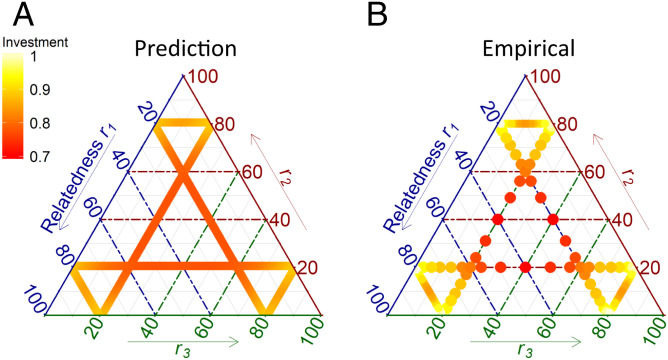
Predicted and observed pattern of stalk investment in three-strain groups. The ternary plot shows the total (collective) level of investment by a group as a function of group composition in terms of the relatedness of the three strains to the group ($r_{1}$,$\;r_{2}$, and $r_{3}$), which is equal to their frequencies in the group). (*A*) The predicted level of collective investment in stalk is indicated by the shading from yellow (high investment) to red (low investment). (*B*) Empirically measured investment in groups across a range of compositions, where each circle indicates the mean investment (calculated from *n* = 20 replicates) by groups with a given composition (where relative investment is inferred from the numbers of spores produced by clonal relative to chimeric groups). Shading matches that for *A*.

To understand how the motivation to contribute to stalk production changes with relatedness (and thus the threat of the tragedy), we examined stalk investment in three-strain groups. This allows a broader range of relatedness patterns to be explored than using pairwise groupings, where the frequencies of the two strains are always counterbalanced. The three-strain groups were constructed over a range of patterns of relatedness where we held the relatedness of one strain constant while varying the relatedness of the others (see Fig. 2*A*). Our approach (both here and in the analysis of multistrain groups described below) assumes that all strains are unrelated (so all pairwise relatedness values are 0). This assumption implies that all strains can distinguish all other strains from self and treat them equally as nonself.

Investment into the stalk was inferred from changes in numbers of spores produced by chimeric aggregations relative to that expected based on the numbers produced when clonal (see *Materials and Methods* for a discussion of the rationale for and caveats associated with the use of relative spore numbers as a means of inferring stalk investment). As expected, we see a decline in stalk investment as relatedness declines in groups (Fig. 2*B*). The best fit of these data from three-strain groups to the Collective Investment game (adapted to allow for biological error in the estimated level of relatedness inferred by individuals; see *Materials and Methods*) occurs when the benefits from stalk investment far exceed the costs (*b* = 12 and *c* = 1, which makes the optimal level of investment, *θ* = 0.46). Experimental measures of collective investment in three-strain groups show a relatively close match to that expected (*r* = 0.75 between model prediction and experimental measures), suggesting that the model provides robust predictions, at least for three-strain groups (Fig. 2*B*). The very high benefit relative to the cost suggests that strains should still be incentivized to invest into stalk production even as relatedness declines well below the levels examined in the three strain groups. However, we would expect that, when relatedness drops below ∼0.1 it will be too low to incentivize any level of investment, and hence collective investment should be 0.

**Fig. 3.**
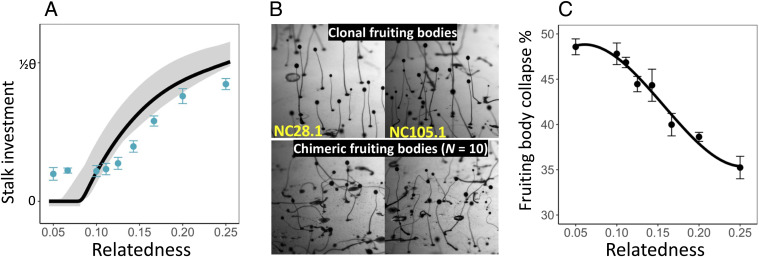
The pattern of collective stalk investment and associated fruiting body stability as a function of relatedness. All *N* strains in a group have equal relatedness (= 1/*N*). (*A*) Stalk investment inferred from the number of spores produced by groups with different compositions relative to that expected when clonal. Points and error bars represent means ± one SE from replicated experiments (average *n* = 22 per group) using different combinations of naturally cooccurring strains. The solid line is the pattern predicted using the best-fit model parameters from three-strain groups and the gray shaded area the 95% confidence interval. Empirical estimates of stalk investment are scaled so that they represent proportions of the model predicted optimal level of investment (*θ*). (*B*) Representative images of fruiting bodies from clonal development and from low relatedness mixes (for mixes of 10 strains at equal frequency). Images are of aggregations generated for the smFISH experiment (and so do not directly correspond to the ones measured in *A* or *B*). (*C*) The proportion of fruiting bodies that spontaneously collapsed (after 48 h) as a function of the relatedness of strains within a group. Individual points and error bars are means ± SE from replicates (average *n* = 11.25 per group) using different groupings of natural strains. The solid line is included for visualization and was fitted by polynomial regression using relatedness cubed.

To examine the impact of the tragedy in groups with lower levels of relatedness than can be achieved in three-strain mixes, and hence determine whether they ultimately suffer catastrophic collapse of stalk investment as predicted, we created groups across a wide range of relatedness levels. For this we mixed sets of *N* strains in equal proportions, such that relatedness of all strains to the group equals 1/*N* (and declines as more strains are added to the groups). As predicted, collective investment deteriorates as relatedness declines but, while we see the expected plateau of collective investment at very low relatedness, investment does not fall to the expected catastrophic level of zero stalk investment, where groups would be unable to take advantage of stalk-facilitated dispersal to escape environments devoid of resources (Fig. 3*A*). Indeed, despite expectations to the contrary, it is clear that very low-relatedness groups still build complete fruiting bodies (with stalks), even if their structure is heavily compromised by their suboptimal stalk investment (Fig. 3*B*). Quantification of fruiting body integrity reveals that the proportion of fruiting bodies that spontaneously collapse increases as relatedness declines (linear model: *F*_1,88_ = 103.5, *P* < 10^−15^) (Fig. 3*B*), which is almost perfectly correlated with the level of collective investment (Pearson correlation: *r* = −0.98, *P* < 10^−4^). These results suggest the presence of a mechanism that enforces contributions to stalk production despite a lack of necessary motivation to invest, which allows groups to avert the expected catastrophic outcome.

Investment of cells into stalk production versus spore formation is ultimately a developmental cell-fate decision. Stalk/spore fate choice in *D. discoideum* is a well-studied model for understanding basic mechanisms governing cell-fate choice and proportioning (32–37). Hence, this knowledge can provide clear hypotheses for how enforcement could arise under conditions where strategic investment results in skewed stalk/spore proportions. Indeed, *D. discoideum* has been shown to possesses a negative feedback mechanism that can buffer clonal development against initial errors in stalk:spore proportioning (32, 34, 35) and which also provides an explanation for how slugs with extreme perturbations in cell type proportioning adjust cell proportions (36–39). The mechanism is based on the idea that differentiation of precursors of stalk cells (prestalk cells) is induced by factors that are produced by the precursors of spore cells (prespore cells) and broken down by the prestalk cells (34, 38, 39). Consequently, if the proportion of prespore cells increases, then the level of stalk-inducing factors will rise (due to increased production and insufficient breakdown), causing some prespore cells to transdifferentiate into prespore cells. Similarly, high prestalk proportions will result in insufficient production of stalk-inducing factors to maintain prestalk cell differentiation, resulting in transdifferentiation into prespore cells. The production and breakdown of these factors is expected to reach an equilibrium around the optimal prestalk:prespore ratio. We hypothesized that this mechanism could also prevent aggregations with very low overall relatedness from showing the predicted zero investment into stalk. In these chimeras, the severe underrepresentation of prestalk cells (owing to a dearth of cells initially committing to the stalk cell fate) would be expected to cause stalk-inducing factor levels to rise significantly (due to increased production and decreased breakdown), which would force some cells to switch from the prespore to the prestalk cell fate. This provides a form of enforcement that would occur as overall relatedness declines, which would increase in importance under the conditions where groups are most at risk for the collapsing tragedy.

**Fig. 4.**
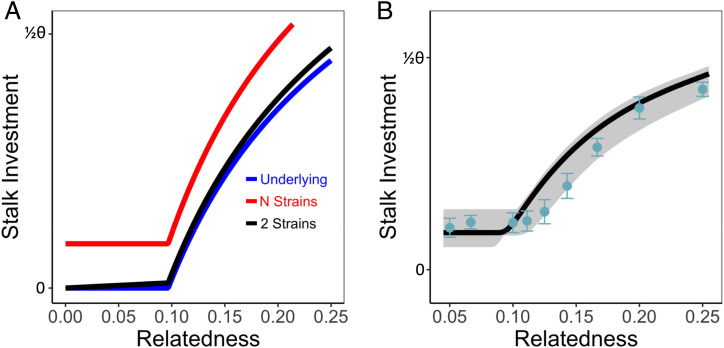
Patterns of stalk investment in the presence of by-product enforcement caused by transdifferentiation. (*A*) The pattern of investment predicted by the model in the absence of transdifferentiation and with transdifferentiation in groups composed of either two strains or *N* strains (where all *N* strains are at equal frequency, making the relatedness of each to the group 1/*N*). Note that, when a strain has very low relatedness to the group, the level of stalk investment they make is much lower when they are in a two-strain group (where their partner strain necessarily has high relatedness) than in groups containing *N* strains (where all *N* have the same low relatedness). (*B*) The empirically measured level of collective investment (where data points match those shown in Fig. 3) with the model predicted investment for the best fit measure of transdifferentiation (where *D* = 0.15) where the remaining model parameters correspond to those fitted without transdifferentiation (see Fig. 3*A*).

To test this idea, we first incorporated a transdifferentiation effect into the Collective Investment game as a pressure on stalk investment that increases as aggregations deviate from the optimal level of investment. The strength of this effect, denoted *D*, is proportional to how far the aggregation is from the optimal stalk–spore proportioning (*θ*). Hence, the presence of transdifferentiation has relatively little effect on investment when groups have high relatedness because most cells in the group will be motivated to invest in stalk and, consequently, the group will not deviate greatly from the optimal level of investment (Fig. 4*A*). However, when groups approach the level of overall relatedness where they would show zero investment, strains are “forced” to invest some proportion of their cells into stalk, despite lacking motivation to do so. As a result, groups can avert the collapsing tragedy even when the strategic response to the low level of relatedness would be zero investment. More generally, the realized pattern of collective investment will depend on the composition of the group since that determines both the inherent level of investment into stalk made by each strain and also the level of transdifferentiation pressure experienced by members of the group. To test this hypothesis, we refitted the model to the data from groups containing different strains at equal frequency (i.e., the data shown in Fig. 3*A*) with the addition of the transdifferentiation parameter *D*. For this, we constrained all other model parameters and simply added the parameter *D* since this approach would ensure that the model still fits the three-strain pattern (since transdifferentiation has a relatively small impact on observed investment in three-strain groups; see Fig. 4*A*). We find that the addition of the transdifferentiation parameter *D* greatly improves the fit of the model to the data (where the model without *D* explains 50% of variation in average stalk production across groups containing different numbers of strains, corresponding to the model predictions in Fig. 3*A*, while the model with *D* explains 89% of the variation, corresponding to the model predictions in Fig. 4*B*). Importantly, the addition of transdifferentiation predicts the plateau in investment below a relatedness of ∼0.1 that is above a realized investment level of 0, whereas the naïve model without transdifferentiation predicts that the plateau represents a value of zero investment.

**Fig. 5.**
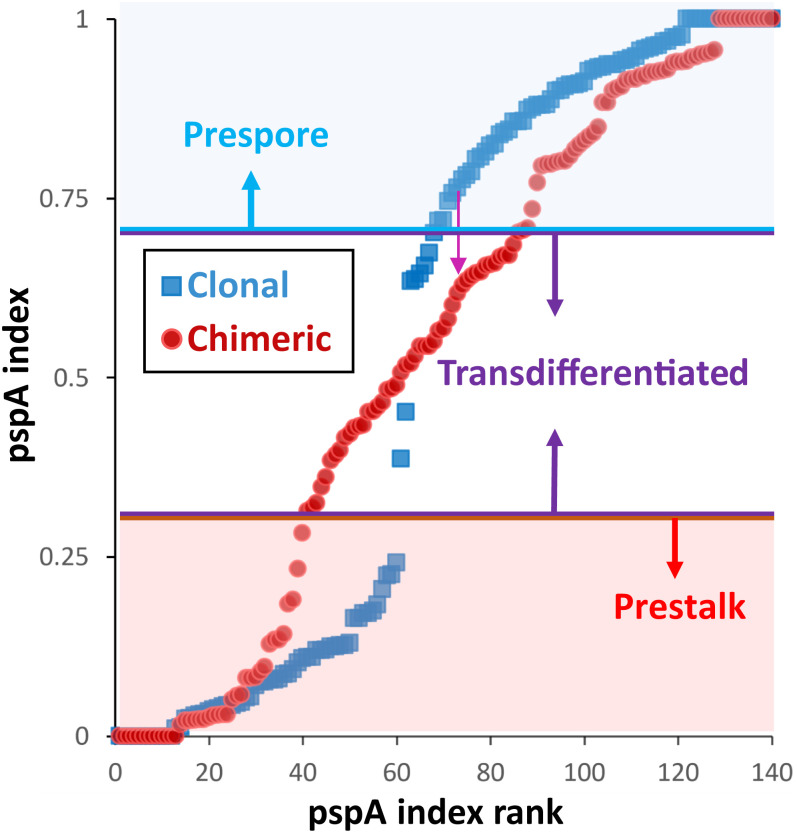
The signatures of transdifferentiation in clonal and chimeric aggregations from smFISH. The pspA index provides a measure of the proportion of expression associated with prespore cell fate. Points represent individual cells from both experimental replicates, which are a mixture of the 10 different genotypes listed in the text. The cells are ranked based on their pspA index to illustrate the overall distribution of index values. Index values below 0.3 are shaded as having a prestalk cell fate and those above 0.7 as having a prespore cell fate. Those in between show mixed cell-fate signatures associated with transdifferentiation. Very few cells in clonal mixes (*n* = 140 cells) show a signature of transdifferentiation, whereas a large proportion of cells from chimeric mixtures (*n* = 154 cells) show clear signatures of transdifferentiation.

Next, we used two complementary approaches to experimentally test the hypothesis that transdifferentiation allows groups to avoid the catastrophic tragedy of zero investment at low relatedness. First, we tested whether strains at very low relatedness show higher investment when in a complex group, where all other strains have low relatedness to the group, than when in a group with one other strain (see Fig. 4*A*). This test reflects the model prediction that, when a strain has low relatedness in a group containing only two strains, it will experience very little pressure for transdifferentiation because its partner strain in the group necessarily has higher relatedness to the group, and so invests close to the optimal level. As a result, a strain with the same low relatedness level should be more able to “defect” by withholding a contribution to the stalk under the conditions where the group is least threatened by the tragedy of the commons (when it is with one other strain), while being forced to invest more when groups are most at risk for the tragedy. To test this prediction, we compared the average level of investment of strains, each at a frequency of 0.05 in groups composed of 20 strains, all at the same frequency, and by strains at a frequency of 0.05 in two-strain groups (where the other strain therefore has a frequency of 0.95). As predicted, we find that strains show significantly lower stalk investment when in two-strain groups than in 20-strain groups (*t*_3_ = 3.64, *P* = 0.036). Second, we used single-molecule fluorescence in situ hybridization (smFISH) to test whether strains in chimeric aggregations show molecular signatures of transdifferentiation. Previous studies have used smFISH probes to the *pspA* and *ecmA* genes to examine prespore and prestalk cell differentiation, respectively (40). In clonal aggregations, expression of these two genes can be used to accurately assign cells to these cell-fate populations since cells show almost no overlap in expression. However, if cells transdifferentiate, then they would be expected to transiently express both markers. We thus took this approach to compare the expression of these genes in clonal and chimeric aggregations of naturally cooccurring strains. For this, strains were developed clonally or in a mix with 8 to 10 other strains until just before slug formation (14.5 h). Clonal and chimeric developmental timing was indistinguishable (*SI Appendix*, Fig. S2). Cells were dissociated, fixed, and hybridized with *ecmA* and *pspA* smFISH probes, and the number of transcripts of each gene was counted in individual cells. Cells were assigned to the prestalk, prespore, or transdifferentiated classes based on an index that captures the relative expression of each gene. The clonal measure of prestalk:prespore proportioning from smFISH gives a prestalk proportion of 43% in clones, which is very close to the predicted optimal level of investment given by the best fit of the data based on spore counts to the model (which is 46%; see above). Given that we expect to see an average level of stalk investment in clones that is near the optimum, this close match between these estimates based on two very different methods further supports the fit of the model to the data and the validity of our methods for inferring stalk investment based on spore production. Furthermore, as expected, clonal aggregations show very low levels of transdifferentiation (Fig. 5) regardless of the thresholds used to define these groups, which is consistent with previous findings. As predicted, chimeric aggregations show significantly higher levels of transdifferentiation compared with clonal aggregations (29% of cells in chimeras, compared with ∼4% in clones: $\chi_{1}^{2}$ = 26, *P* < 10^−6^). There are also significantly fewer prestalk cells in chimeras relative to clones (43% in clones versus 26% in chimeras, $\chi_{1}^{2}$ = 6.3, *P* = 0.012), which is consistent with the observation that chimeric groups show reduced stalk production. This pattern is consistent with the idea that a high proportion of cells in chimeras initially adopt the prespore cell fate and then expresses prestalk markers (and eventually adopt stalk-cell fate) as the pressure to transdifferentiate influences cell-type-specific gene expression. Interestingly, our inference that the proportion of cells allocated to the stalk is *ca*. 43 to 46% (based both on spore counts and markers of cell fate) is higher than the values frequently mentioned in the literature [which are typically in the 20 to 30% range (41–43)]. Inspection of the sources of these previous estimates reveals that they are almost entirely based on many independent measurements from a single widely used strain (NC-4) or strains derived from it (e.g., AX4). Measurement of stalk:spore proportioning in an array of natural isolates (44, 45) has demonstrated that strains vary in their proportioning and that NC-4 (which is equivalent to the strain NC96.1 that was used in these studies) shows well-below-average stalk proportioning (and at a level consistent with its difference from the average proportioning we report here). Therefore, to confirm the accuracy of our estimated stalk proportioning relative to the widely reported estimates from the NC-4 related strains, we carried out the same smFISH experiment on clonal aggregations of AX4, yielding an overall estimate of 29% of cells showing prestalk cell fate (versus prespore cell fate), which is in line with the estimates in the literature.

Our finding that the underlying mechanisms controlling realized contributions to cooperation can act to constrain selfish cheating and enforce contributions to group-beneficial public goods has important implications for understanding the evolution of cooperative traits, including the origin of complex cells, multicellularity, and societies. The emergence of mechanisms that ensure high relatedness or that enforce cooperation between unrelated individuals is considered fundamental for these major evolutionary transitions (13, 46). In our study, we show that transdifferentiation acts to enforce cooperation in low relatedness groups, promoting enhanced cooperation under the conditions where individuals are expected to lack motivation to contribute to cooperation. However, we suggest that this is an inadvertent by-product (13) of selection on clonal cell proportioning that has evolved independently from its effect on cooperation. In this scenario, selection for optimal fruiting body architecture shapes the central mechanism of cell-fate signaling to allow it to buffer clonal groups against perturbations in cell-type proportioning [e.g., from variation in the energetic states of cells at the time of aggregation (32, 34)], which could otherwise threaten the success of the group. Consistent with this idea, we see very little transdifferentiation in normal clonal development where allocation to stalk versus spore is presumably close to optimal, whereas forced transdifferentiation markedly increases as a by-product of perturbations in proportioning within chimeras. Furthermore, although enforcement from transdifferentiation would affect fitness of chimeras, providing a potential source of selection, the expected effect it would have on the fitness of high- and low-relatedness members of a group makes it logically consistent with it being a by-product of selection in clonal development, not chimeric development. This is because its effect on fitness is opposite to what would be expected for it to have evolved as an adaptive mechanism to enforce cooperation in chimeric groups. The strains that disproportionally produce enforcement (i.e., the stalk-inducing factors that lead to transdifferentiation) are the ones at the lowest relatedness in the group (and hence have the highest prespore initial cell fate), but they are the members of the group that least benefit from being enforced. At the same time, group members at high relatedness, who would most benefit from enforcement, are those who are providing the least enforcement pressure (and given their higher initial commitment to prestalk cell fate, they are actually actively reducing the enforcement pressure by breaking down the stalk-inducing factors).

Thus, while we see widespread tuning of strategic investment in chimeras (see also ref. 15), our results strongly suggest that patterns of strategic investment are constrained by transdifferentiation, which presumably evolved due to selection for developmental canalization to ensure appropriate stalk:spore proportioning in clones. The presence of transdifferentiation can, thus, be viewed as imposing a functional constraint (47) on the developmental system that prevents genotypes from implementing a stalk investment strategy that would maximize their success across all contexts. For example, when a strain has very low relatedness to the group, the dispersal benefit of sacrificing a proportion of its cells to provide a tiny incremental increase in stalk size will almost certainly not counter the direct fitness cost in terms of lost potential spore production, making zero investment the best strategy under these conditions. However, the presence of transdifferentiation can prevent the strain from actually achieving this strategy (even if their initial cell fate decision follows this optimal pattern), and hence it acts as a constraint on the realization of that strategy. In this way, this enforcement mechanism may be analogous to the origin of worker policing in social insects (48, 49), where workers consume other workers’ eggs. Worker policing ultimately increases altruism, but the trait likely evolved as a consequence of the selfish benefits of workers consuming eggs they are not highly related to, rather than because of its effect on the level of altruism (50). Such similarities highlight the hitherto unappreciated role of by-product enforcement in the stabilization of cooperation.

## Materials and Methods

### The Collective Investment Game.

We use the Collective Investment game to model strategic cooperation through public goods. The full model is outlined in ref. 18 and so is only briefly described here. Players in the game are considered to be competing genetic variants that interact in groups, making their relatedness to the group ($r_{i}$) equal to the frequency of their particular variant in the group. Given their relatedness to the group, players strategically invest a portion of their resource budget ($x_{i}$) into public goods, while their residual budget ($1-x_{i}$) determines a direct fitness component. Each unit of investment incurs a cost of c to their direct fitness and generates a benefit to the group of $r_{i}b$ (where the player’s relatedness to the group dictates the degree to which their contribution is diluted as a group-level benefit). Setting the total budget that individuals have to invest in public goods at 1 (such that *x_i_* is constrained to be ≥0 and ≤1), makes the total personal cost $C_{i}=1-cx_{i}$ and group-level benefit $B_{i}=1+r_{i}bx_{i}$. The costs and benefits form a trade-off between investment in personal fitness (survival and/or fecundity) and group benefit (which raises personal fitness by enhancing survival and/or fecundity), making the two multiplicative (hence fitness, $\omega_{i}=\;B_{i}C_{i}$). For example, in *D. discoideum*, dispersal benefits arising from production of the stalk (the public good) are realized via the spores produced (24, 25), so any contribution to public goods means that a strain essentially sacrifices their ability to reap those rewards (because they produce fewer spores through which the dispersal benefit can be realized).

The optimal investment in public goods for a player (${\hat{x}}_{i}$) (in terms of the marginal effect its contribution has on its own fitness) as a function of its relatedness to a group can be solved by finding the level of contribution that maximizes fitness with respect to the personal costs and benefits associated with that contribution, which gives

[1] $${\hat{x}}_{i}=\{\begin{array}{cc} \frac{1}{2}\left(\frac{1}{c}-\frac{1}{br_{i}}\right), & \text{if}\;\frac{1}{2}\left(\frac{1}{c}-\frac{1}{br_{i}}\right)>0\\ 0, & \text{otherwise} \end{array},$$

where the conditional statement captures the fact that investment has to be nonnegative. The relationship between Eq. **1** and Hamilton’s rule (10) can be seen by examining the threshold between contributing and not contributing to stalk formation (i.e., the threshold given by the condition in Eq. **1**), which occurs when $r_{i}b-c>0$, meaning players should stop investment when relatedness drops below *c*/*b*. The sum of the individual contributions from all players in a group gives the total collective investment for the group: $x_{G}=\sum_{i=1}^{N}{\hat{x}}_{i}r_{i}$ (note $r_{i}$ represents the frequency weighting in the summation since the whole group relatedness for a player is equal to its frequency in the group). Groups composed of a single player (where $r_{i}$ = 1) will invest at a level (θ) that maximizes the total fitness of the group, which is given by (b−c)/2bc. Therefore, we can evaluate the extent of the tragedy of the commons by considering how far groups are from the level of investment into public goods that maximizes group success (i.e., the distance between θ and $x_{G}$), and likewise can evaluate how individual players undermine potential for group success by evaluating how far their individual investment is from this value (i.e., the distance between θ and $x_{i}$).

### Information and Error.

The Collective Investment game implicitly assumes that players have perfect information about their relatedness to the group, but in biological systems organisms will almost certainly make some degree of error in estimating their relatedness to the group. This error could reflect the fact that individuals are only able to sample information from a limited number of group members, or it could reflect noisiness in the signals or in the system used to assess the signals that provide information on relatedness. The nonlinear relationship between optimal investment and relatedness means that the presence of error is expected to alter the pattern of investment, which will therefore alter the fit of the model to empirical data. We include error by assuming that error in the estimation of relatedness by the cells from a given strain follows a Gaussian probability density function (PDF), where the mean of the PDF represents their true relatedness (meaning that the assessment of their relatedness is correct, on average, but shows error among cells within a group) and the SD represents the level of noisiness among cells. Because group complexity and the nature of information can change systematically with relatedness, we also allow for frequency-dependent error (where a strain’s frequency in a group is equal to its relatedness to the group). For this we allow the level of nosiness to change with frequency of a strain in the group such that the SD of the error function is weighted by $4^{t}{\left[r_{i}(1-r_{i})\right]}^{t}$, where again $r_{i}$ is the relatedness of the strain to the group, while the exponent *t* indicates the degree of frequency dependence (i.e., if *t* = 0, then there is no frequency dependence, while increasing values of *t* above 1 increasingly concentrate error at intermediate levels of relatedness) (*SI Appendix*, Fig. S3). This function is based on the logic that signals are likely more reliable if relatedness is very low or approaches 1, whereas it is likely to be more difficult to distinguish between different possible levels of intermediate relatedness. Error is incorporated by calculating the average expected investment by a strain using numerical iteration over all levels of relatedness for each value of true relatedness. Cells from a given strain are assumed to make an investment decision that follows the optimal pattern (given by Eq. **1**), but which is based on their independent measure of relatedness (which reflects error), rather than their true relatedness.

### Robustness to Model Assumptions.

The Collective Investment game assumes that benefits from public goods are a linear function of investment. However, benefits could potentially be nonlinear. Therefore, we evaluated the robustness of the main model predictions to nonlinearity of benefits by using two general shapes of nonlinear benefit functions: diminishing and accelerating returns (*SI Appendix*, *Supplementary Information Text*). Importantly, we find that these different functions do not alter the qualitative pattern of investment by strains, but rather they shift the expected level of investment above or below that expected from the linear function (*SI Appendix*, Fig. S4). Because these patterns are analogous to those expected from the linear benefits function with a different value of benefits and costs (compare *SI Appendix*, Fig. S4 with Fig. 1), the model based on the assumption of linearity is used for fitting empirical data.

### Transdifferentiation and By-Product Enforcement.

To consider how negative feedback associated with transdifferentiation changes patterns of investment we incorporate a biologically motivated component into the Collective Investment game. For this we make a critical distinction between the initial allocation of cells to stalk made a given strain (*A_i_*), which is based on the strain’s relatedness to the group (*r_i_*), and the realized investment of cells to stalk (*x_i_*, which matches the symbol used for the model without transdifferentiation to reflect the fact that it is the realized allocation to stalk that determines fitness in both cases), the latter of which depends on how the initial allocation of cells is modified by transdifferentiation. We refer to the initial decision as “inherent investment” since it represents the actual strategy being invoked by the player and the latter as “realized investment” since it represents the level actually shown (which is what determines fitness). We assume that the mechanism of transdifferentiation arises from a negative feedback system that is at equilibrium if the group makes an initial allocation of cells to stalk that matches the level that optimizes group fitness, *θ* (which implies the system has evolved as a mechanism of developmental buffering to ensure adaptive allocation of cells in clonal groups). This assumption matches evidence from clonal groups, where the proportion of cells showing molecular signatures of transdifferentiation is near 0 (40). As the proportion of cells allocated to the stalk by the group deviates from *θ* (meaning inherent investment is below optimal investment) the group experiences a pressure for transdifferentiation determined by the parameter *D*. Hence, the total pressure from transdifferentiation is *D*(*θ* − *A_G_*), which makes the allocation of cells to stalk by a given strain equal to *x_i_* = *A_i_* + *D*(*θ* − *A_G_*), meaning that a strain will be pushed to allocate more cells to the stalk than their initial allocation whenever the group allocates less than the optimal level (i.e., whenever *θ* > *A_G_*).

We assume that natural selection shapes *A_i_* at each level of relatedness to the group to maximize the player’s fitness. Because fitness depends on realized investment (*x_i_*), the optimal initial allocation of cells to stalk (${\hat{A}}_{i}$) will depend on the total allocation of cells to stalk by the entire group (*A_G_*, because it determines the pressure for transdifferentiation), which depends on the expected (i.e., average) group composition. For simplicity we assume that patterns of investment have evolved in groups containing large numbers of strains, which means that the initial allocation of cells to stalk for all nonfocal strains is 0. The pattern of expected initial allocation for this scenario is largely the same as that under the other extreme, which is groups containing only two strains (so the relatedness of the nonfocal strain is 1 – *r_i_*). Under this scenario, the optimal initial investment is

[2] $${\hat{A}}_{i}(r_{i})=\{\begin{array}{cc} \frac{1}{2}\left(\frac{1-D}{c(1-Dr_{i})}-\frac{1}{br_{i}}\right), & \text{if}\;\frac{1}{2}\left(\frac{1-D}{c(1-Dr_{i})}-\frac{1}{br_{i}}\right)>0\\ 0, & \text{otherwise} \end{array}$$

(where $r_{i}$ logically is bounded between 0 and 1). This expression generally means that the presence of transdifferentiation will reduce the initial allocation of cells to stalk when $r_{i}$ < 1 (compared with the model without transdifferentiation; i.e., where *D* = 0) because transdifferentiation makes the realized investment in stalk higher than the initial allocation of cells to stalk whenever groups are chimeric (and hence the initial allocation to stalk would be expected to evolve to be lower to compensate). Transdifferentiation then transforms the value of ${\hat{A}}_{i}(r_{i})$ into a realized value, and fitness is determined as in the case with no transdifferentiation (above).

### Empirical Methods.

We tested model predictions using a set of 24 naturally occurring strains of *D. discoideum* from North Carolina (NC), which have previously been used in many studies (17, 44, 45): NC28.1, NC34.1, NC34.2, NC39.1, NC43.1, NC52.3, NC54.2, NC58.1, NC60.1, NC60.2, NC63.2, NC67.2, NC69.1, NC71.1, NC73.1, NC76.1, NC78.2, NC80.1, NC85.2, NC87.1, NC88.2, NC96.1, NC99.1, and NC105.1.

### Manipulation of Group Composition.

In the first set of experiments we created groups containing three different strains. Because there is such a huge array of possible frequency combinations that can be constructed from sets of three strains, we explored frequency space by varying frequencies along “transects” through this space, where each strain was held constant at a frequency of either 0.2 or 0.8 while the frequencies of two other strains in the group were varied across a set of 10 frequency combinations. This yielded a total of 60 unique frequency combinations for a set of three strains (which represent 20 different frequency combinations, but with each of three strains in a triplet being treated as the focal strain while the other two were varied gives 60 different combinations). These frequency combinations are indicated by the positions of the data points in Fig. 1 across the three-strain frequency space. Each frequency combination was replicated an average of 21 times, for a total of 420 measurements of collective investment in three-strain groups. Each experimental replicate also included replicates of clonal development. Clonal development for each strain was independently replicated an average of 10.25 times, with each independent replicate including an average of 2.9 technical replicates, for a total of 239 separate measures (see Dataset S1 for details on the frequencies used in the mixes and the strain IDs).

In the second set of experiments, we explored a wider range of relatedness values by increasing the number of strains in each group. For this we created groups of *N* strains (where *N* was 4, 5, 6, 7, 8, 9, 10, 15, or 20) in which all strains were at a frequency of 1/*N* in the group, which means that the average relatedness in each group is 1/*N*. Each of the nine frequency combinations was replicated an average of 22 times, for a total of *n* = 198 measurements of collective investment in *N*-strain groups. As with the three-strain group experiment, each experimental replicate also included replicates of clonal development. Clonal development for each strain was independently replicated an average of 3 times, with each independent replicate including an average of 2.7 technical replicates, for a total of 165 measures (see Dataset S2 for details on replication and group composition).

### Measurement of Spore Production.

The protocols for quantifying spore allocation in *D. discoideum* are well documented (26, 44) and so described only briefly here. Strains were grown on *Klebsiella aerogenes* as a food source. After growth, amoebae were harvested and washed by centrifugation in KK2 buffer (16.1 mM KH_2_PO_4_ and 3.7 mM K_2_HPO_4_). Amoebae were then counted on a hemocytometer and resuspended in KK2 at a density of 10^8^ cells per mL. Chimeric or clonal groups were created by adding cells from each strain at the relevant relative frequency in a 1.5-mL Eppendorf and mixing thoroughly. Cells (10^7^) of each mix were then spread evenly on a 6-cm Petri dish containing 1.5% nutrient-free agar in KK2 and left to develop for 24 h in an incubator at 22 °C. For collective investment measures (see below), all fruiting bodies were harvested in 5 mL of spore buffer and counted on a hemocytometer. The measurements of total number of spores in chimeric ($T_{G}$) and clonal ($T_{i}$) groups were used infer stalk investment in chimeric groups relative to that expected from clones (see below).

### Measurement of Fruiting Body Stability.

To measure fruiting body stability, we created groups of *N* strains following the same approach as described above in which all strains were at a frequency of 1/N in the group, with a total of eight different types of groups being measured (where *N* was 4, 5, 6, 7, 8, 9, 10, or 20). Briefly, cells were deposited on nonnutrient agar within each well of a 24-well dish in 10-µL spots containing 10^8^ cells per mL (so each spot contained ∼10^6^ cells). Plates were left undisturbed for 48 h to allow for spontaneous collapse of fruiting bodies. The percentage of total fruiting bodies within each well that had fallen over (collapsed) was counted directly from plates using a stereomicroscope (17). Each of the eight group types were replicated an average of 11.25 times for a total of 90 measurements of fruiting body collapse (see Dataset S3 for details on replication and group composition). We tested for a relationship between fruiting body collapse and average relatedness (which is equal to 1/*N*, where *N* is the number of strains in a group, with all at equal relatedness) using a mixed model fitted by maximum likelihood, where average relatedness was a fixed effect (as a continuous variable) and the group composition (which identified each unique combination of strains) was a random effect (which controlled for variation in the collective behavior of different strain combinations).

### Estimating Stalk Investment.

Direct quantitative measurement of cell numbers in stalks is not possible due to the nature of stalk cells. Instead, the total level of collective investment in stalk by a chimeric group was estimated using a proxy that compares their production of spores ($T_{G}$) relative to that expected based on spore production by clonal groups. This approach is based on the idea that if strains do not respond to chimerism then a chimera will produce the same number of spores as the weighted average of the constituent strains (where each is weighted by its frequency in the group). This approach is built on the underlying assumption that differences in the number of spores produced by groups that differ in composition/relatedness are caused by shifts in the proportional allocation of cells to stalk versus spores. The success of this approach depends on whether relatedness-dependent changes in spore numbers are indeed primarily caused by changes in stalk:spore proportioning or whether they instead reflect systematic biases that alter the relative number of spores produced. For example, if other phenomena such as the proportion of nonaggregating cells (51–53) or the degree of cell division (45) change systematically with relatedness, then changes in spore numbers would not directly reflect changes in stalk investment. However, there are several lines of evidence that support the validity of using changes in spore numbers to estimate changes in stalk investment. First, to provide direct experimental evidence in support for this approach we used strains transformed with a constitutively expressed RFP reporter gene to track cells in stalk and spore cell tissues in clonal and low relatedness groups (see *SI Appendix*, *Supplementary Information Text* for a description of these methods and information on the associated datasets). By characterizing their distribution in both slugs and fruiting bodies, this approach reveals that, as expected, cells with low relatedness to the group are underrepresented in both the prestalk region of slugs (relative to the pattern in clonal groups; *F*_1,26_ = 146, *P* < 10^−11^) and in fruiting body stalks (*F*_1,19.6_ = 55.3, *P* < 10^−6^) (see also *SI Appendix*, Fig. S1). These findings are thus consistent with the inferences based on more indirect measures that suggest chimeric fruiting bodies allocate more cells to spores. Second, previous studies have shown that the absolute number of spores recovered from fruiting bodies of chimeras composed of strain pairs is higher than for clones of the same strains (44). Similarly, both absolute and relative numbers of spores recovered from chimeric groups (in relation to the production by equivalent clonal groups) increases as overall relatedness declines (17). Finally, the assumption that changes in spore numbers reflect changes in stalk investment is supported by studies that have linked these changes to meaningful variation in fruiting body architecture. They have been shown to correspond to estimates of stalk:spore proportioning derived from fruiting body morphology (44) and are very highly correlated to measures of fruiting body stability (17) (and hence are predictive of apparent functional changes in fruiting body architecture). While it is conceivable that processes other than the relative allocation to stalk and spore fates could lead to higher absolute and relative spore production as a function of differences in relatedness, the parsimonious explanation is arguably that the patterns mostly reflect changes in stalk investment. Thus, this collective body of evidence together supports the use of measures of relative spore production as a means of estimating of stalk investment.

Because strains vary in their clonal level of investment in stalk (44), we estimated the expected spore production for each chimeric group ($E_{G}$) by taking the mean of the clonal values ($T_{i}$) for the constituent strains (where the clonal spore production by strains is weighted by their frequency in the group, $r_{i}$):

[3] $$E_{G}=\overset{N}{\underset{i=1}{\sum }}r_{i}T_{i}.$$

To generate a measure of relative spore production for a chimeric group we divided the value expected based on clonal spore production, $E_{G}$, by the measured spore production of the group, $T_{G}$:

[4] $$S_{G}=\frac{E_{G}}{T_{G}}.$$

Because we expect strains to be investing at a level that corresponds to the optimum (*θ*) when clonal, we infer that, when $S_{G}=1$ strains are showing the optimal level of investment (*θ*), and values less than 1 represent a reduction in stalk investment compared that which is optimal for the group (i.e., compared with the clonal expectation $E_{G}$). Therefore, to relate our empirical measures of investment to the model expectations, we label a value of $S_{G}=1$ as *θ* and scale values <1 as a level of investment ($I_{G}$) given by

[5] $$I_{G}=\frac{\theta -1+S_{G}}{S_{G}}.$$

These values can be expressed as a proportion of *θ* ($y_{i}$) by simply dividing the value in Eq. **5** by the inferred value of *θ*.

### Comparing Empirical Data with Model Predictions.

To allow a direct comparison between model predictions and empirical data, we calculated collective investment from the model using the same method that we used to estimate the measure from empirical data (based on Eq. **4**). For this we need to express collective investment in terms of spore production scaled to the clonal expectation for spore production. Therefore, the predicted collective investment from the model (${\hat{x}}_{G}$) has the expected value (denoted $X_{G}$) of

[6] $$X_{G}=\frac{1-\theta }{\left(1-{\hat{x}}_{G}\right)}$$

when rescaled to match the empirical methods. To plot empirical results along with model predictions on a meaningful scale, we generated a predicted value of θ from the model and used it in Eq. **5** to calculate the inferred level of investment ($I_{G}$), which is then plotted in the same space as the model predictions based on $X_{G}$.

### Comparing Investment in Two-Strain Groups with 20-Strain Groups.

We compared the level of collective investment made by 20-strain groups with the level made by individual strains in two-strain groups when at a frequency of 5%. In both cases, this provides a measure of investment that strains make when they have a relatedness to their group of 5%, but with the background level of relatedness differing between these group types. That is, because each individual strain in a 20-strain group is at the same 5% frequency, collective investment represents the average individual investment of the constituent strains (and hence separate measurements of individual strains are not needed). The dataset for two-strain groups contains measures of investment for three different strains that show a frequency-dependent response to chimerism (each replicated seven independent times) from ref. 17. This set was measured using the same experimental conditions as the data from the 20-strain groups, making the two comparable (Dataset S4). To account for among-pair variation caused by systematic differences in numbers of spores produced by different strains (see ref. 44), we adjusted the representation of each strain the pairwise chimeric mixes to account for the overall difference in their representation in 50:50 mixes, which were done at the same time. The relative representation of strains in the 50:50 mixes allows us to identify the systematic overrepresentation of either strain in the pairwise mix, which could bias the estimate of investment at 5% if unaccounted for. These two measures of investment were compared using a mixed model, where replicate ID was a random effect (corresponding to either the strain ID for two-strain groups, or N for 20-strain groups) and group type (two-strain versus 20-strain) was a fixed effect. To account for the data structure, we enforced the minimal degrees of freedom on the significance test (based on the number of replicate groups).

### Molecular Analysis of Signature of Transdifferentiation.

To identify molecular signatures of transdifferentiation we created clonal and chimeric groups and used an approach based on FISH to quantify markers of prespore and prestalk cell fate. For clonal development, we used 10 different strains (NC28.1, NC34.2, NC52.3, NC60.1, NC63.2, NC69.1, NC71.1, NC80.1, NC99.1, and NC105.1) that were independently cultured and allowed to develop on separate agar plates. For chimeric development, equal quantities of these 10 strains were mixed and allowed to develop. All plates were harvested after 14.5 h of development, when all were at the early slug stage of development. Structures from individual plates were dissociated into single cells in KK2/20 mM ethylenediaminetetraacetic acid (EDTA) buffer by passing through a 25-gauge needle several times. Cells were washed with KK2 twice and resuspended in KK2 at a cell density of 2 × 10^6^ cells per mL. For clonal developments, cells from each clone were mixed in equal proportions to generate a pool with the same representation of each strain as from chimeric development. Cells were fixed on a coverslip by treatment with 3.7% formaldehyde/phosphate-buffered saline (PBS) for 10 min. Fixed cells were washed twice with PBS and stored in 70% of ethanol at 4 °C before performing single-molecule hybridization chain reaction (smHCR) using the Multiplexed in Situ HCR v3.0 protocol for tissue sections on slides provided by Molecular Technologies (54). Two biological replicates were performed. HCR probe sets and fluorophore-labeled HCR hairpins were purchased from Molecular Technologies. Samples were hybridized overnight with 4 nM of each HCR probe. HCR amplification was performed overnight with 60 nM of each HCR hairpin (conjugated to Alexa 488 or Alexa 594 to visualize pspA and ecmA messenger RNA transcripts, respectively). For the first biological replicate, 19 separate images of cells from clonal development were taken, containing a total of 253 individuals cells, and 12 images were taken of cells from chimeric development containing a total of 217 individual cells. In the second replicate, four images were taken from clonal development (*n* = 112 individual cells) and four images were taken from chimeric development (*n* = 110 individual cells). We obtained the number of dots of Alexa 488 and Alexa 594 signals in individual cells using FISH-quant software (55). Cells with fewer than 17 dots from either gene were excluded from analyses because low counts give ambiguous cell fate assignments. After censoring, we normalize the expression level of pspA to that of ecmA in the individual cells by multiplying the pspA counts by the ratio of the ecmA to pspA mean counts. Data from the separate replicates were normalized independently to account for differences in the efficiency of the hybridization in the two experiments. This produced a set of individual cell counts for pspA and ecmA with the same mean (which arbitrarily corresponds to the mean of ecmA), allowing us to use the two sets of counts to construct an index that indicated the relative expression of the two genes in each cell. Using the normalized dots number of pspA and raw dots number for ecmA, we calculate this pspA index for each cell as pspA^norm.^/(ecmA + pspA ^norm.^), which indicates the proportion of all dots that were from pspA. Cells with a pspA index ≤0.3 were classified as prestalk, those with an index ≥0.7 were classified as prepore, and all cells in between these thresholds were considered to be transdifferentiated. Proportions of cells in each of these categories (prespore, prestalk, and transdifferentiated) in clonal vs. chimeric groups were compared independently using χ^2^ tests with one degree of freedom. The independent replicates of the experiment yielded very similar proportions of cells in the three categories across the clonal replicates ($\chi_{2}^{2}$ = 1.66, *P* = 0.44), across the chimeric replicates ($\chi_{2}^{2}$ = 2.29, *P* = 0.32), and across the two replicates overall ($\chi_{2}^{2}$ = 3.95, *P* = 0.41).

These methods were also used to estimate prestalk:prespore proportioning and the incidence of transdifferentiation in clonal groups of the commonly used AX4 laboratory strain. Two replicates were performed, one in which plates were harvested after 14 h (*n* = 161 cells) and one in which plates were harvested after 16 h (*n* = 156 cells). The two replicates yielded almost identical proportions of cells in the prespore, prestalk, and transdifferentiated categories ($\chi_{2}^{2}$ = 1.96, *P* = 0.37).

### Fitting the Collective Investment Game to Data.

We used a process of numerical iteration to identify the set of parameters (*b*, *c*, *e*, and *f*) that provided the best fit of the model to the average stalk investment for each composition of three-strain groups. To find the best fit model, we first defined a large search-space of the four variables, *b*, *c*, *e*, and *f* (2≤b≤10;1≤c<2;0≤e≤1; 0<f≤1), with 20 values chosen for each parameter. For each unique combination of *b*, *c*, *e*, and *f*, we then derived the optimal strategy (Eq. **6**) for each combination of *b*, *c*, *e*, and *f*. We then used our approach to modeling error (see *Information and Error*) to calculate the optimal strategy with error. Individual investment from the model was converted to predicted collective investment on the empirical scale (Eq. **6**) corresponding to each of the unique social scenarios (relative relatedness of all players) for which we had empirical data.

For model fits when using the model that allows transdifferentiation through the parameter *D*, we took the best-fit parameters from the model without transdifferentiation and used an iterative approach to search for the value of *D* (in intervals of 0.01 such that *D* ≥ 0 and *D* ≤ 0.25) that gives the best fit to empirical data. For each possible value of *D*, we calculated the optimal strategy (Eq. **2**) and converted it to a value comparable to empirical data (Eq. **6**). We then compared the fit of the model prediction with empirical data.

The fit between predictions and the empirical data were always assessed using a least-squares approach. To test the quality of the model fit to data, we used a paired *t* test of each pair of empirical data and corresponding model prediction.

To calculate confidence intervals around the best-fit model we use a resampling approach. Briefly, we took samples of each data point from distributions corresponding to the empirical data and calculated the best-fit parameters using the same approach as above. We then repeated this approach for 100 iterations. To calculate confidence intervals for each parameter, we used the range between the 5th and 95th percentiles of the deviations between the parameter value of the overall best-fit model and the parameter value of each iteration. For plotting, we calculate a single confidence interval around the overall best fit as the combination of upper and lower confidence intervals of all three parameters that gives the greatest deviation in predicted investment from the overall best-fit model. As such, our confidence interval is a conservative estimate of confidence in the true values of the parameters.

To assess the utility of adding another variable, error in relatedness estimation, to the model fit, we first used a broad search space of parameters b and c  and a least-squares approach to fit the perfect information model with the b and c  values that best matched the data. Next, we added another parameter, e, to make an imperfect information model, where e is the SD of the error in measuring relatedness (see *Information and Error*). To test for the significant of difference in fit between perfect and imperfect information models we used an *F* test.

All statistical analysis and data processing was conducted in the statistical program R. Ternary plots for the three-player game were plotted using the ggtern package in R. All other figures were plotted using the package ggplot2 in R.

## Supplementary Material

Supplementary File

Supplementary File

Supplementary File

Supplementary File

Supplementary File

## Data Availability

All data for all experiments are included in the datasets. The dataset corresponding to each experiment is noted in *Materials and Methods* at the point where the experimental design is described. The R code used for fitting the Collective Investment game to data is available on GitHub via github.com/lauriebelch/tragedy_of_the_commons (56). All other study data are included in the article and/or supporting information.

## References

[1] A. F. G. Bourke, Principles of Social Evolution (Oxford University Press, 2011).

[2] S. A. West, A. S. Griffin, A. Gardner, Evolutionary explanations for cooperation. Curr. Biol. 17, R661–R672 (2007). 17714660 10.1016/j.cub.2007.06.004

[3] D. J. Rankin, K. Bargum, H. Kokko, The tragedy of the commons in evolutionary biology. Trends Ecol. Evol. 22, 643–651 (2007). 17981363 10.1016/j.tree.2007.07.009

[4] M. Olson, The Logic of Collective Action (Harvard University Press, 1965).

[5] G. Hardin, The tragedy of the commons. Science (80-.) 162, 1243–1248 (1968). 5699198

[6] H. S. Gordon, The economic theory of a common-property resource: The fishery. J. Polit. Econ. 62, 124–142 (1954).

[7] E. Ostrom, Governing the Commons: The Evolution of Institutions for Collective Action (Cambridge University Press, 1990).

[8] T. Wenseleers, F. L. W. Ratnieks, Tragedy of the commons in Melipona bees. Proc. Biol. Sci. 271 (suppl. 5), S310–S312 (2004). 15504003 10.1098/rsbl.2003.0159 PMC1810046

[9] J. E. Strassmann, D. C. Queller, Privatization and property in biology. Anim. Behav. 92, 305–311 (2014).

[10] W. D. Hamilton, The genetical evolution of social behaviour. I. J. Theor. Biol. 7, 1–16 (1964). 5875341 10.1016/0022-5193(64)90038-4

[11] A. Dragoš , Division of labor during biofilm matrix production. Curr. Biol. 28, 1903–1913.e5 (2018). 29887307 10.1016/j.cub.2018.04.046 PMC6331042

[12] C. Simonet, L. McNally, Kin selection explains the evolution of cooperation in the gut microbiota. Proc. Natl. Acad. Sci. U.S.A. 118, e2016046118 (2021). 33526674 10.1073/pnas.2016046118 PMC8017935

[13] J. A. Ågren, N. G. Davies, K. R. Foster, Enforcement is central to the evolution of cooperation. Nat. Ecol. Evol. 3, 1018–1029 (2019). 31239554 10.1038/s41559-019-0907-1

[14] K. R. Foster, T. Wenseleers, F. L. W. Ratnieks, Kin selection is the key to altruism. Trends Ecol. Evol. 21, 57–60 (2006). 16701471 10.1016/j.tree.2005.11.020

[15] P. D. Taylor, G. Wild, A. Gardner, Direct fitness or inclusive fitness: How shall we model kin selection? J. Evol. Biol. 20, 301–309 (2007). 17210023 10.1111/j.1420-9101.2006.01196.x

[16] A. Gardner, S. A. West, Inclusive fitness: 50 years on. Philos. Trans. R. Soc. Lond. B Biol. Sci. 369, 20130356 (2014). 24686928 10.1098/rstb.2013.0356 PMC3982658

[17] P. G. Madgwick, B. Stewart, L. J. Belcher, C. R. L. Thompson, J. B. Wolf, Strategic investment explains patterns of cooperation and cheating in a microbe. Proc. Natl. Acad. Sci. U.S.A. 115, E4823–E4832 (2018). 29735672 10.1073/pnas.1716087115 PMC6003509

[18] P. G. Madgwick, J. B. Wolf, Evolution of strategic cooperation. Evol. Lett. 4, 164–175 (2020). 32313691 10.1002/evl3.164 PMC7156107

[19] S. A. Frank, Mutual policing and repression of competition in the evolution of cooperative groups. Nature 377, 520–522 (1995). 7566147 10.1038/377520a0

[20] K. R. Foster, G. Shaulsky, J. E. Strassmann, D. C. Queller, C. R. L. Thompson, Pleiotropy as a mechanism to stabilize cooperation. Nature 431, 693–696 (2004). 15470429 10.1038/nature02894

[21] S. A. West, T. E. Kiers, I. Pen, R. F. Denison, Sanctions and mutualism stability: When should less beneficial mutualists be tolerated? J. Evol. Biol. 15, 830–837 (2002).

[22] F. L. W. Ratnieks, P. K. Visscher, Worker policing in the honeybee. Nature 342, 796–797 (1989).

[23] O. Pellmyr, C. J. Huth, Evolutionary stability of mutualism between yuccas and yucaa moths. Nature 372, 257–260 (1994).

[24] J. E. Strassmann, Y. Zhu, D. C. Queller, Altruism and social cheating in the social amoeba Dictyostelium discoideum. Nature 408, 965–967 (2000). 11140681 10.1038/35050087

[25] J. Smith, D. C. Queller, J. E. Strassmann, Fruiting bodies of the social amoeba Dictyostelium discoideum increase spore transport by *Drosophila*. BMC Evol. Biol. 14, 105 (2014). 24884856 10.1186/1471-2148-14-105 PMC4038703

[26] R. H. Kessin, Dictyostelium: Evolution, Cell Biology, and the Development of Multicellularity (Cambridge University Press, 2001).

[27] O. M. Gilbert, K. R. Foster, N. J. Mehdiabadi, J. E. Strassmann, D. C. Queller, High relatedness maintains multicellular cooperation in a social amoeba by controlling cheater mutants. Proc. Natl. Acad. Sci. U.S.A. 104, 8913–8917 (2007). 17496139 10.1073/pnas.0702723104 PMC1885602

[28] C. H. Waddington, Canalization of development and the inheritance of acquired characters. Nature 150, 563–565 (1942).

[29] E. A. Ostrowski, M. Katoh, G. Shaulsky, D. C. Queller, J. E. Strassmann, Kin discrimination increases with genetic distance in a social amoeba. PLoS Biol. 6, e287 (2008). 19067487 10.1371/journal.pbio.0060287 PMC2586364

[30] R. Benabentos , Polymorphic members of the lag gene family mediate kin discrimination in Dictyostelium. Curr. Biol. 19, 567–572 (2009). 19285397 10.1016/j.cub.2009.02.037 PMC2694408

[31] N. Gruenheit , A polychromatic ‘greenbeard’ locus determines patterns of cooperation in a social amoeba. Nat. Commun. 8, 14171 (2017). 28120827 10.1038/ncomms14171 PMC5288501

[32] R. R. Kay, P. Flatman, C. R. L. Thompson, DIF signalling and cell fate. Semin. Cell Dev. Biol. 10, 577–585 (1999). 10706822 10.1006/scdb.1999.0341

[33] K. Uchinomiya, Y. Iwasa, Evolution of stalk/spore ratio in a social amoeba: Cell-to-cell interaction via a signaling chemical shaped by cheating risk. J. Theor. Biol. 336, 110–118 (2013). 23911583 10.1016/j.jtbi.2013.07.024

[34] C. R. L. Thompson, R. R. Kay, Cell-fate choice in Dictyostelium: Intrinsic biases modulate sensitivity to DIF signaling. Dev. Biol. 227, 56–64 (2000). 11076676 10.1006/dbio.2000.9877

[35] R. R. Kay, C. R. L. Thompson, Cross-induction of cell types in Dictyostelium: Evidence that DIF-1 is made by prespore cells. Development 128, 4959–4966 (2001). 11748133 10.1242/dev.128.24.4959

[36] R. Pogge‐von Strandmann, R. R. Kay, Position‐dependent regulation of the prestalk‐prespore pattern in Dictyostelium slugs. Dev. Genet. 11, 447–452 (1990).

[37] K. Mohri, R. Tanaka, S. Nagano, Live cell imaging of cell movement and transdifferentiation during regeneration of an amputated multicellular body of the social amoeba Dictyostelium discoideum. Dev. Biol. 457, 140–149 (2020). 31563450 10.1016/j.ydbio.2019.09.014

[38] R. R. Kay, S. Large, D. Traynor, O. Nayler, A localized differentiation-inducing-factor sink in the front of the Dictyostelium slug. Proc. Natl. Acad. Sci. U.S.A. 90, 487–491 (1993). 8421680 10.1073/pnas.90.2.487 PMC45688

[39] C. R. L. Thompson, R. R. Kay, The role of DIF-1 signaling in Dictyostelium development. Mol. Cell 6, 1509–1514 (2000). 11163223 10.1016/s1097-2765(00)00147-7

[40] V. Antolović, T. Lenn, A. Miermont, J. R. Chubb, Transition state dynamics during a stochastic fate choice. Development 146, dev173740 (2019). 30890571 10.1242/dev.173740 PMC6602359

[41] M. Hayashi, I. Takeuchi, Quantitative studies on cell differentiation during morphogenesis of the cellular slime mold Dictyostelium discoideum. Dev. Biol. 50, 302–309 (1976). 945203 10.1016/0012-1606(76)90153-6

[42] D. R. Garrod, I. M. Ashworth, Effect of growth conditions on development of the cellular slime mould, Dictyostelium discoideum. J. Embryol. Exp. Morphol. 28, 463–479 (1972). 4674569

[43] I. Takeuchi, M. Hayashi, M. Tasaka, “Cell differentiation and pattern formation in dictyostelium” in Development and Differentiation in the Cellular Slime Moulds, P. Cappuccinelli, J. M. Ashworth, Eds. (Elsevier, 1977), pp. 1–16.

[44] N. J. Buttery, D. E. Rozen, J. B. Wolf, C. R. L. Thompson, Quantification of social behavior in D. discoideum reveals complex fixed and facultative strategies. Curr. Biol. 19, 1373–1377 (2009). 19631539 10.1016/j.cub.2009.06.058

[45] J. B. Wolf , Fitness trade-offs result in the illusion of social success. Curr. Biol. 25, 1086–1090 (2015). 25819562 10.1016/j.cub.2015.02.061 PMC4406944

[46] S. A. West, R. M. Fisher, A. Gardner, E. T. Kiers, Major evolutionary transitions in individuality. Proc. Natl. Acad. Sci. U.S.A. 112, 10112–10119 (2015). 25964342 10.1073/pnas.1421402112 PMC4547252

[47] T. F. Hansen, “Evolutionary constraints” in Oxford Bibliographies in Evolutionary Biology, J. Losos, Ed. (Oxford University Press, 2015), pp. 1–28.

[48] A. Griffin, Policing. Curr. Biol. 29, R431–R432 (2019). 31163151 10.1016/j.cub.2019.03.064

[49] F. L. W. Ratnieks, K. R. Foster, T. Wenseleers, Conflict resolution in insect societies. Annu. Rev. Entomol. 51, 581–608 (2006). 16332224 10.1146/annurev.ento.51.110104.151003

[50] T. Wenseleers, F. L. W. Ratnieks, Enforced altruism in insect societies Cooperation. Nat. Rev. Microbiol. 444, 6–9 (2006). 10.1038/444050a 17080081

[51] D. Dubravcic, M. Van Baalen, C. Nizak, R. Gomer, An evolutionarily significant unicellular strategy in response to starvation in Dictyostelium social amoebae. F1000Research 3, 133 (2014). 25309731 10.12688/f1000research.4218.1 PMC4184345

[52] C. E. Tarnita, A. Washburne, R. Martinez-Garcia, A. E. Sgro, S. A. Levin, Fitness tradeoffs between spores and nonaggregating cells can explain the coexistence of diverse genotypes in cellular slime molds. Proc. Natl. Acad. Sci. U.S.A. 112, 2776–2781 (2015). 25605926 10.1073/pnas.1424242112 PMC4352809

[53] F. W. Rossine, R. Martinez-Garcia, A. E. Sgro, T. Gregor, C. E. Tarnita, Eco-evolutionary significance of “loners”. PLoS Biol. 18, e3000642 (2020). 32191693 10.1371/journal.pbio.3000642 PMC7081983

[54] M. Dunagin, M. N. Cabili, L. Rinn, A. Raj, “Visualization of lncRNA by single-molecule fluorescence in situ hybridization” in Nuclear Bodies and Noncoding RNAs, S. Nakagawa, T. Hirose, Eds. (*Methods in Molecular Biology*, Humana Press, 2015), pp. 3–19. 10.1007/978-1-4939-2253-6_1 25555572

[55] F. Mueller , FISH-quant: Automatic counting of transcripts in 3D FISH images. Nat. Methods 10, 277–278 (2013). 23538861 10.1038/nmeth.2406

[56] L. J. Belcher ., Code for analysis of the collective investment game for the manuscript “Developmental constraints enforce altruism and avert the tragedy of the commons in a social microbe”. GitHub. https://github.com/lauriebelch/tragedy_of_the_commons. Deposited 28 June 2022. 10.1073/pnas.2111233119 PMC9303850 35858311

