# Supplementary material for: Developmental constraints enforce altruism and avert the tragedy of the commons in a social microbe
Source: Proc Natl Acad Sci U S A. 2022 Jul 15;119(29):e2111233119. doi: 10.1073/pnas.2111233119 (PMC9303850; doi:10.1073/pnas.2111233119)
Supplement: Supplementary File [file pnas.2111233119.sapp.pdf]

PNAS

Supplementary Information for
Developmental constraints enforce altruism and avert the tragedy of the commons in a social microbe

Laurence J. Belcher[1], Philip G. Madgwick[1], Satoshi Kuwana[2], Balint Stewart[2], Christopher R. L. Thompson[2*] and Jason B. Wolf[1*]

*Jason B. Wolf and Christopher R. L. Thompson
Email: j.b.wolf@bath.ac.uk, christopher.thompson@ucl.ac.uk

This PDF file includes:

Supplementary text
Figures S1 to S4
SI References

Other supplementary materials for this manuscript include the following:

Datasets S1 to S7

Supplementary Information Text

Cell tracking in clonal and chimeric slugs and fruiting bodies using RFP labelling

For visualisation and quantification of cell fate allocation in clonal and chimeric fruiting bodies, cells from the strains NC52.3, NC60.1,NC63.2, NC80.1 NC99.1, and NC105.1 were prepared for transformation according to ref. (1). Cells were transformed with the plasmid pDM1210 which allows cells to be marked by constitutive expression of mCherry. In parallel, cells were transformed with the pDM1029 empty vector, which serves as a control for transformation and selection. Following selection in the presence 10µg/ml G418, cells were harvested and developed in mixes of 10% RFP expressing and 90% mock transfected cells. Clonal and chimeric mixtures were created following the same protocol as outlined in the main text (see the section *Measurement of Spore Production* in the *Materials and Methods*). For chimeric groups, RFP expressing cells from one strain were mixed with mock transfected cells from a different strain, producing groups where labelled cells had low relatedness to the group (with relatedness being 0.1). For clonal groups, both cell types were derived from the same strain. Slugs and fruiting bodies were imaged by fluorescence microscopy under mineral oil and quantification of stalk fluorescence was performed using Fiji software. One slug and one fruiting body from each clonal and chimeric mixture was used to generate a measure of relative fluorescence intensity that captures the distribution of labelled cells. For fruiting bodies, fluorescence intensity was measured throughout the entire stalk by manually tracing around the stalk (replicated three times and normalised to stalk area). Background fluorescence was measured by taking the average of three regions of the stalk where fluorescent cells were not visible (normalised to area). Normalised background fluorescence was subtracted from the normalised stalk fluorescence to provide a measure of the degree to which stalks contain RFP labelled cells. If cells respond to low relatedness by shifting their cell fate towards spores then we would expect stalks in chimeric fruiting bodies to have lower relative fluorescence than those from clonal fruiting bodies (because the labelled cells in chimeras have low relatedness to the group). Because fruiting bodies varied in the degree of fruiting body maturation, we scored the fruiting bodies on a scale from 1 to 3 to capture this variation (where a score of 3 represents a mature or very nearly mature fruiting body) and tested for a difference using chi-square. For slugs, fluorescence intensity was measured in the anterior quarter, which provides an estimate of the fluorescence in the prestalk region, and in the posterior three-quarters, providing an estimate of the fluorescence of the prespore region. For this, the length of each slug was first estimated by manually fitting a line from the anterior to posterior extremes, which was then used to divide the slug into these two sections. We then manually traced around each section and measured total fluorescence intensity (normalised to area). The difference in the normalised fluorescence intensity of the prestalk and prespore regions (measured as prespore minus prestalk) was calculated for each slug. Similar to the case for stalks, if cells respond to low relatedness by shifting their cell fate towards spores, we would expect the prespore:prestalk fluorescence difference to be higher in chimeric than in clonal slugs (because the low-relatedness labelled cells in chimeras would be shifted towards the prespore region). The measures of relative fluorescence intensity in clonal and chimeric mixtures were compared separately for stalks and slugs using a mixed model with aggregation type (clonal or chimeric) as a fixed effect and the labelled genotype as a random effect (after square-root transformation, with negative values replaced by zero). Denominator degrees of freedom were adjusted based on the random effect using the Kenward-Rogers approximation.

Representative images of clonal and chimeric fruiting bodies and slugs are shown in Figure S1 (with the full set included in Datasets S6 and S7). In both fruiting bodies and slugs, we see clear qualitative patterns that follow the expected reduction in stalk investment at low relatedness. For the fruiting bodies, we see an obvious qualitative shift in cell fate, with labelled cells being underrepresented in the stalks of low relatedness groups. Likewise, in slugs we see an obvious qualitative difference between clonal and chimeric groups, with labelled cells being underrepresented in the prestalk region. These observations are supported by the quantitative measurements. Relative fluorescence intensity of stalks in clonal fruiting bodies is significantly higher than in chimeric fruiting bodies ($F_{1,\,19.8}$ = 55.32, p < 0.0001), reflecting the fact that cells from the lower relatedness strain are underrepresented in the stalk. This pattern is not caused by a difference in fruiting body maturity between clonal and chimeric groups (χ^2_2 = 1.39, *p* = 0.50). A corresponding pattern is seen in slugs where the prespore:prestalk fluorescence difference of chimeric groups is significantly higher than that of clonal groups ($F_{1,\,26}$ = 146, p < 0.0001). Although the relationship between fluorescence and RFP labelled cell density is potentially non-linear (since the relative fluorescence presumably does not change linearly with density of fluorescent cells owing to saturation), we can also examine the qualitative match between the pattern inferred from the RFP fluorescence measures and the pattern of clonal and chimeric stalk investment previously inferred from spore count data in two-strain groups with the same relatedness (using data from ref. 2). The ratio of average stalk fluorescence of the two groups (about 4.8:1) is relatively close to the ratio of clonal to chimeric stalk allocation estimated previously (which is about 4:1). Likewise, the average ratio of prespore to prestalk fluorescence in chimeric slugs (~0.33) is a close match to the ratio (~0.34) that would be expected based on the previously estimated relative increase in spore production by strains at the same relatedness in two-strain chimeras. Hence, direct tracking of cells in clonal and chimeric fruiting bodies reveals the expected shift in allocation of cells to the stalk as predicted from measures based on spore counts in these same strains.

Robustness to model assumptions

We evaluate the robustness of the main model predictions to non-linearity of benefits using two general shapes of non-linear benefit functions: diminishing and accelerating returns (see Figure S4). For each, we derive a new function for group benefits and solve the optimal investment. The non-linear equations for group benefits B_G are as follows: diminishing returns $B_G = 1 + b[1 - (1 - x_G)^{1.3}]$ and accelerating returns $B_G = 1 + b{x_G}^{1.3}$, where x_G is the investment of the group. Importantly, these different functions do not alter the qualitative pattern of investment by strains, but rather, they shift the expected level of investment above or below that expected from the linear function.

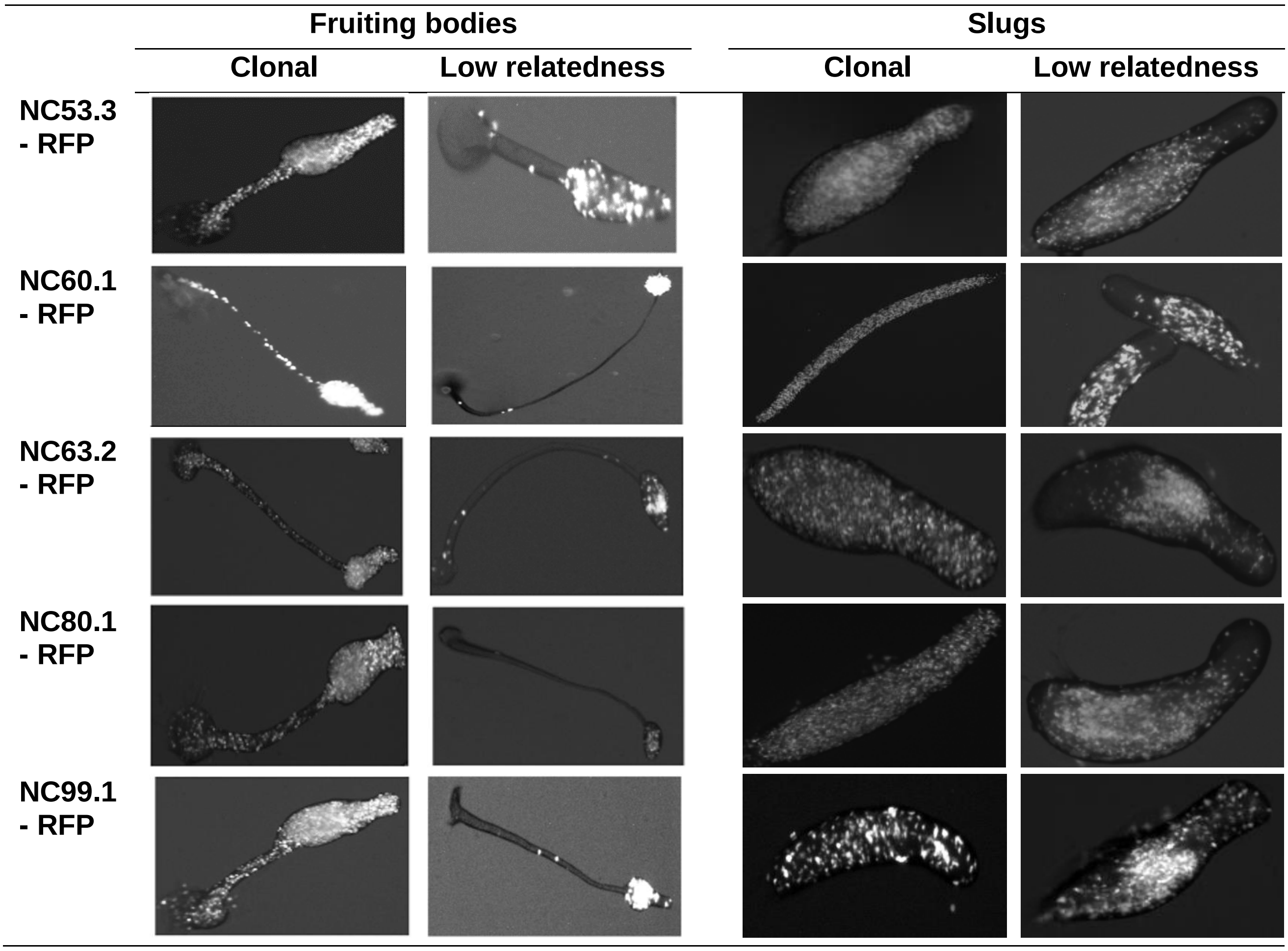

Figure S1. Representative images of clonal and chimeric fruiting bodies and slugs containing RFP-expressing and mock-transformed control cells. Each row contains images of groups containing the strain listed in the first column (NC52.3, NC60.1,NC63.2, NC80.1 and NC99.1) either as a clonal group or as the low relatedness labelled strain in a chimeric mixture. For the first four strains (NC52.3, NC60.1,NC63.2, and NC80.1) the low relatedness mixtures contain RFP-expressing cells of that strain at a frequency of 10% and mock-transformed cells of the strain NC99.1 at a frequency of 90%. For the row containing NC99.1, the low relatedness mixtures contain RFP-expressing NC99.1 at a frequency of 10% and mock-transformed cells from NC52.3 at a frequency of 90%. For fruiting bodies, the stalks of low relatedness mixtures show lower levels of fluorescence than the clonal mixtures. For slugs, the prespore region (anterior quarter) of low relatedness slugs shows lower fluorescence (relative to the posterior three-quarters) than the clonal mixtures (which show an even distribution of fluorescently labelled cells throughout).

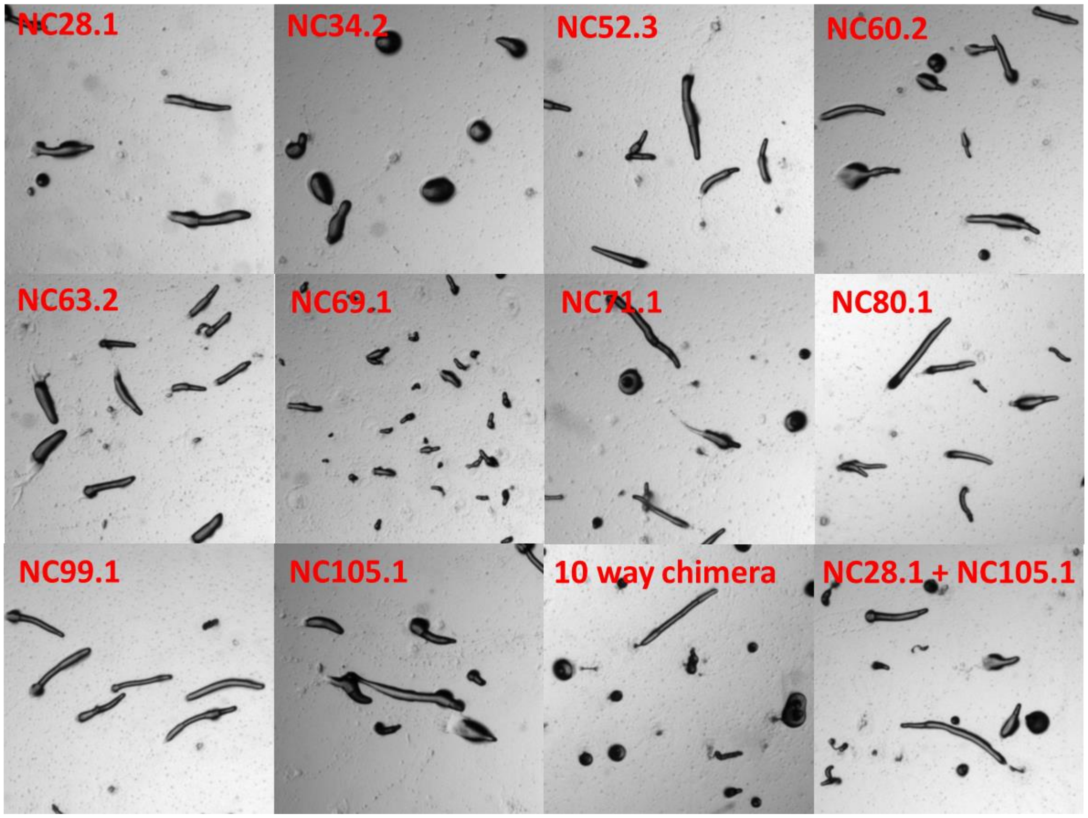

Figure S2. Images of aggregations used in the smFISH experiment. All images were taken at 14.5 hours into development. Each image labelled with a single strain ID represents a plate with clonal aggregations composed of that strain. The 10-way chimera is composed of a mix of these ten strains, each in equal proportion. We also include an image of a two-way chimera composed of one of the strain pairs for comparison but did not use pairwise mixes in the smFISH experiment.

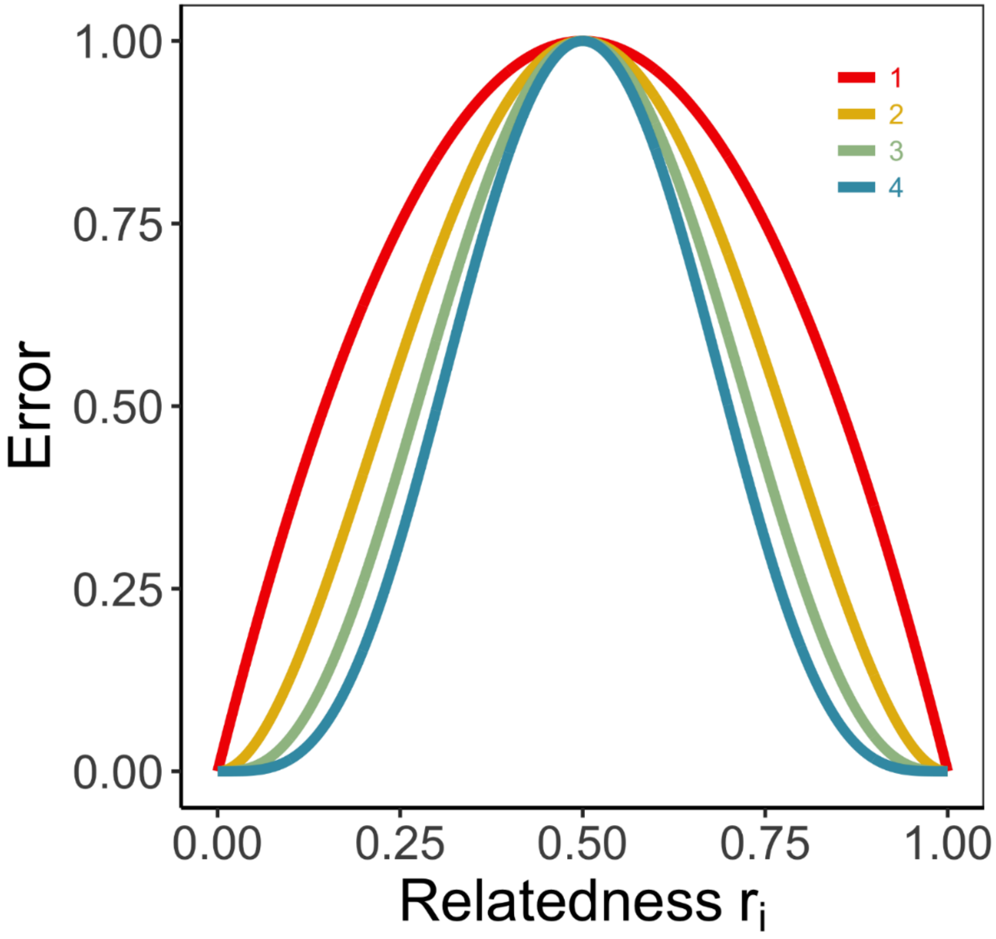

Figure S3. The shape of frequency dependent error. The different lines represent different exponents of the error function, with a higher exponent corresponding to a strong degree of frequency dependence in error. Each line is defined by the equation $4^t[r_i(1 - r_i)]^t$, where the lines corresponds to the value of t and the x-axis to the values of r_i.

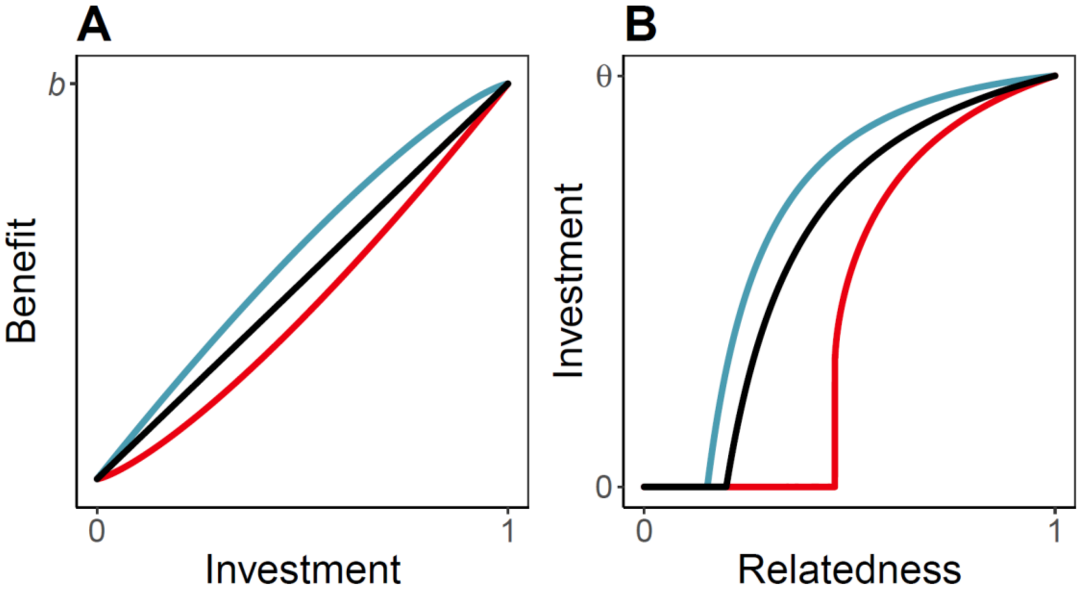

Figure S4. The impact of non-linearity on patterns of strategic investment. **A**) the three lines show a linear relationship between investment and benefits from public goods (which is assumed in the Collective Investment Game) as well as diminishing and accelerating benefits. **B**) expected patterns of strategic investment as a function of relatedness for linear, diminishing, and accelerating benefits.

Dataset S1 (separate file) Investment data for three strain group. This file contains data from groups composed of three strains mixed at varying proportions and data from clonal development for the same set of strains. The first sheet contains the data from chimeric mixes: the strain IDs and frequencies in the mix, the observed number of spores for the chimera, the number of spores produced by each strain in clonal development, the expected number of spores (based on the weighted average of the spore production by the constituent strains when clonal), and the inferred level of collective investment. The second sheet contains the data from clonal development for the same set of strains. The column of means are values averaged over technical replicates.

Dataset S2 (separate file) Investment data for N strain groups. This file contains data from groups composed of *N* strains, with each at a proportion of 1/*N*, and data from clonal development for the same set of strains. The first sheet contains the data from chimeric mixes: the strain IDs for each mix, the observed number of spores for the chimera, the number of spores produced by each strain in clonal development, the expected number of spores (based on the weighted average of the spore production by the constituent strains when clonal), and the inferred level of collective investment. The second sheet contains the data from clonal development for the same set of strains. The column of means are values averaged over technical replicates.

Dataset S3 (separate file) Fruiting body collapse data. This file contains data from groups composed of *N* strains, with each at a proportion of 1/*N*. Each row represents data from one plate containing N strains, with the set of strains indicated in the strain compositions columns: the total number of fruiting bodies on the plate, the number of collapsed fruiting bodies, and the percent collapsed.

Dataset S4 (separate file) Investment data for two and 20 strain groups. The file contains measurements of stalk investment for strains at a frequency of 5% in two-strain mixes and for chimeras composed of a set of 20 strains mixed at equal frequencies.

Dataset S5 (separate file) smFISH data. The file contains data on the dot counts from the smFISH experiment in clonal and chimeric mixtures for a set of natural strains and clonal data from the lab strain AX4. The first sheet contains data from clonal development: the experiment ID (1 or 2), the imagine number, the block ID, dot counts for ecmA and pspA, these values after censoring for low counts, the rescaled value of pspA, and the pspA index. The second sheet contains this same information for the chimeric mixes. The third sheet contains the same data for clonal development in AX4.

Dataset S6 (separate file) Fruiting body images and stalk fluorescence measures. The file contains images of fruiting bodies under fluorescence for RFP and associated measurements of stall fluorescence. Images are arranged in rows corresponding to the RFP labelled strain at a frequency of 0.1 and the columns the mock-transformed cells at a frequency of 0.9. Below each image there is a set of three repeats of the measurement of the total stalk fluorescence and three separate measures of fluorescence in areas without RFP expressing cells. The final column in each set has the means, with the difference in means below. These measures are summarized on the sheet with the processed data, which also includes an estimate of the relative maturation level of the fruiting body.

Dataset S7 (separate file) Slug images and prestalk_prespore fluorescence measures. The file contains images of slugs under fluorescence for RFP and associated measurements of prestalk and prespore fluorescence. Images are arranged in rows corresponding to the RFP labelled strain at a frequency of 0.1 and the columns the mock-transformed cells at a frequency of 0.9. Below each image there is a set of three repeats of the measurement of the fluorescence of the prespore and prestalk regions. The final column in each set has the means, with the difference in means below. These measures are summarized on the sheet with the processed data, which also includes the proportional fluorescence of the prestalk region.

SI References

1. Paschke P, et al. (2018) Rapid and efficient genetic engineering of both wild type and axenic strains of Dictyostelium discoideum. *PLoS One* 13(5):e0196809.
2. Madgwick PG, et al. (2018) Strategic investment explains patterns of cooperation and cheating in a microbe. *Proc Natl Acad Sci* 115(21):E4823–E4832.
